# The red/far-red light photoreceptor FvePhyB regulates tissue elongation and anthocyanin accumulation in woodland strawberry

**DOI:** 10.1093/hr/uhad232

**Published:** 2023-11-17

**Authors:** Qi Gao, Shaoqiang Hu, Xiaoli Wang, Fu Han, Huifeng Luo, Zhongchi Liu, Chunying Kang

**Affiliations:** National Key Laboratory for Germplasm Innovation & Utilization of Horticultural Crops, Huazhong Agricultural University, Wuhan, 430070, China; Hubei Hongshan Laboratory, Wuhan, 430070, China; National Key Laboratory for Germplasm Innovation & Utilization of Horticultural Crops, Huazhong Agricultural University, Wuhan, 430070, China; Hubei Hongshan Laboratory, Wuhan, 430070, China; National Key Laboratory for Germplasm Innovation & Utilization of Horticultural Crops, Huazhong Agricultural University, Wuhan, 430070, China; Hubei Hongshan Laboratory, Wuhan, 430070, China; National Key Laboratory for Germplasm Innovation & Utilization of Horticultural Crops, Huazhong Agricultural University, Wuhan, 430070, China; Hubei Hongshan Laboratory, Wuhan, 430070, China; Institute of Horticulture, Hangzhou Academy of Agricultural Sciences, Hangzhou, 310024, China; Department of Cell Biology and Molecular Genetics, University of Maryland, College Park, MD, 20742, USA; National Key Laboratory for Germplasm Innovation & Utilization of Horticultural Crops, Huazhong Agricultural University, Wuhan, 430070, China; Hubei Hongshan Laboratory, Wuhan, 430070, China

## Abstract

Light is an important environmental signal that influences plant growth and development. Among the photoreceptors, phytochromes can sense red/far-red light to coordinate various biological processes. However, their functions in strawberry are not yet known. In this study, we identified an EMS mutant, named P8, in woodland strawberry (*Fragaria vesca*) that showed greatly increased plant height and reduced anthocyanin content. Mapping-by-sequencing revealed that the causal mutation in *FvePhyB* leads to premature termination of translation. The light treatment assay revealed that FvePhyB is a bona fide red/far-red light photoreceptor, as it specifically inhibits hypocotyl length under red light. Transcriptome analysis showed that the *FvePhyB* mutation affects the expression levels of genes involved in hormone synthesis and signaling and anthocyanin biosynthesis in petioles and fruits. The *srl* mutant with a longer internode is caused by a mutation in the DELLA gene *FveRGA1* (*Repressor of GA1*) in the gibberellin pathway. We found that the P8 *srl* double mutant has much longer internodes than *srl*, suggesting a synergistic role of *FvePhyB* and *FveRGA1* in this process. Taken together, these results demonstrate the important role of *FvePhyB* in regulating plant architecture and anthocyanin content in woodland strawberry.

## Introduction

Light is not only an important source of energy, but also acts as an environmental signal throughout the plant life cycle, which can influence plant morphology and adaptability to the environment. Light signals can be classified as light intensity, light quality, photoperiod and direction of light, which are perceived by different photoreceptors. Photoreceptors are divided into three main categories according to their absorption spectrum: UV-B receptor UV Resistance locus 8 (UVR8), blue light receptors cryptochromes (CRY) and phototropins (PHOT), and red/far-red light receptors phytochromes (PHY) [1–3]. Phytochromes are unique in that they have the red light-absorbing form (Pr) and the far red light-absorbing form (Pfr) that are distinct but photoreversible. The inactive Pr form can absorb red light (with a maximum value at 660 nm) and convert to the Pfr form, while the active Pfr form can absorb far-red light (with a maximum value at 730 nm) and convert to the Pr form or revert to Pr in darkness [4]. Phytochromes are localized in the cytosol in the dark and translocated to the nucleus in the light [5–7]. Recent studies have shown that PhyB is also a temperature sensor that regulates plant responses to changes of external temperature [8, 9].

Phytochromes are grouped into a small gene family that is relatively conserved in different plant species [10]. There are five phytochrome genes in *Arabidopsis thaliana*, namely *phytochrome A* (*PhyA*), *PhyB*, *PhyC*, *PhyD*, and *PhyE*. The PhyA protein is light stable, whereas the others are all light labile [11]. The active phytochromes would interact with the bHLH transcription factors phytochrome interacting factors (PIFs) and cause their phosphorylation and degradation [12]. Alternatively, phytochromes interact with the E3 ubiquitin ligase constitutive photomorphogenic 1 (COP1), thereby reducing the degradation of the bZIP transcription factor HY5 (long hypocotyl 5), the bHLH transcription factor HFR1 (long hypocotyl in far-red 1), or the MYB transcription factor LAF1 (Long After Far-red light 1) [13].

A prominent function of *PhyB* is to regulate organ elongation in plants. In doing so, it interacts directly or indirectly with several hormone signaling pathways. For example, PhyB interacts directly with the auxin pathway transcription factors ARF6 (auxin response factors 6) and ARF8 in a red light-dependent manner to inhibit hypocotyl elongation in Arabidopsis [14]. DELLA proteins as key repressors in the GA pathway regulate both the sequestration and degradation of PIFs, a way to coordinate light and GA activities during hypocotyl elongation [15–17]. Light signals can modulate the stability of BZR1 (Brassinazole-Resistant 1), a key regulatory factor in the brassinolide (BR) signaling pathway, via COP1 to control the formation of active BZR1 homodimer or BZR1-PIF4 heterodimer [18]. PhyB modulates the stability of the cytokinin response regulator ARR4 (Arabidopsis Response Regulator 4) by direct interaction [19].

Light provides an environmental cue to fine-tune anthocyanin accumulation in plant tissues [20]. Anthocyanins are water-soluble flavonoids that give rich colors to various plant organs with antioxidant capacity and nutritional value. The anthocyanin biosynthesis pathway includes a series of structural genes, such as *CHS* (*chalcone synthase*), *CHI* (*chalconeisomerase*), *F3H* (*flavanone3-hydroxylase*), *DFR* (*dihydroflavonol-4-reductase*), *ANS* (*anthocyanidin synthase*), and *UFGT* (*flavonoid3-O-glucosyl-transferase*) [21, 22]. Anthocyanins are first synthesized in the endoplasmic reticulum and then moved to the vacuole for long-term storage via GST (glutathione S-transferase) and MATE (multidrug and toxic compound extrusion) [23]. The MYB transcription factor, the bHLH transcription factor and the WD40 protein form the MBW complex, an important transcriptional regulator of the structural genes in the anthocyanin pathway [24]. In addition, the light signaling components COP1, HY5, and PIFs are also involved in the light-controlled anthocyanin accumulation [25–29].

In strawberry, anthocyanins are the main pigments in the vegetative tissues and mature fruits, regulated by different transcription factors and structural genes [30, 31]. MYB10 (MYB domain protein 10) is a key transcription factor that affects anthocyanin biosynthesis in fruits of both wild and cultivated strawberries [32–35]. However, *FveMYB10L*, which is adjacent to *FveMYB10* with a high similarity, regulates anthocyanin biosynthesis in petioles [36]. The MYB transcription factor genes *FaMYB1/5/9/11/123* also play roles in fruit pigmentation in cultivated strawberry [37–39]. In addition to biosynthesis, the GST gene *RAP* (*reduced anthocyanin in petioles*) plays a crucial role in anthocyanin transport from the cytosol to the vacuole for long-term storage [40, 41]. Although light is an important environmental signal for fine-tuning fruit color in strawberry [29], the contribution of each photoreceptor to anthocyanin levels is not known.

In this study, we have identified an ethyl methanesulfonate (EMS) mutant of *FvePhyB* in the wild diploid strawberry *Fragaria vesca*, which exhibits increased plant height and reduced anthocyanin content. Further analysis revealed that *FvePhyB* is a functional *PhyB* ortholog that mediates photomorphogenesis under red light. *FvePhyB* can regulate the expression of several genes involved in hormone biosynthesis and signal transduction, as well as anthocyanin biosynthesis. In addition, simultaneous mutation of *FvePhyB* and *FveRGA1* changed the growth habit of strawberry from rosette to caulescent. These results elucidate the functions of *FvePhyB* in plant growth in woodland strawberry.

## Results

### The *F. vesca* P8 mutant has reduced anthocyanins in petioles and is much taller than wild type

**Figure 1:**
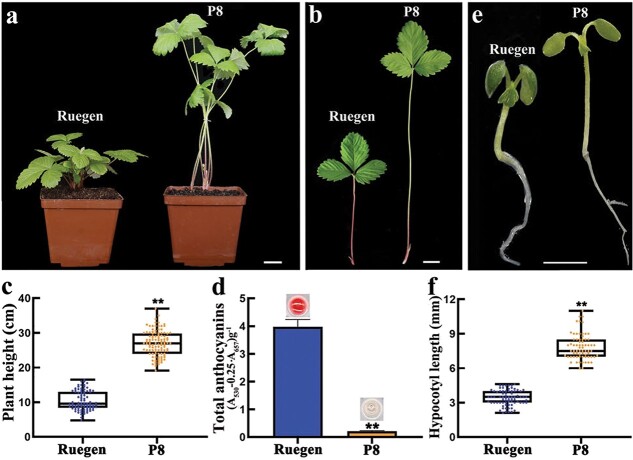
Phenotypes of the EMS mutant P8 in *Fragaria vesca.***a** Whole plant of wild-type Ruegen and the P8 mutant. **b** The leaf of Ruegen and P8. **c** Plant height of Ruegen and P8, *n* ≥ 85. **d** Total anthocyanin contents in the leaf petioles of Ruegen and P8. The upper images show the anthocyanin extract. Data are the mean ± SD of three biological replicates. **e** The seedling of Ruegen and P8. **f** The hypocotyl length of Ruegen and P8, *n* ≥ 69. ^**^P < 0.01, Student’s *t*-test. Scale bars: 2 cm (**a**, **b**), 2 mm (**e**).

In order to identify the regulatory genes of plant growth in strawberry, ethyl methanesulfonate (EMS) mutagenesis was performed on the woodland strawberry variety Ruegen. In the M_2_ population, a mutant called P8 was identified, which is less red and much taller than the wild type (Fig. 1a). Similar to the whole plant, the petioles of P8 are extremely elongated, and the upper part accumulates much less red pigment (Fig. 1b). Measurements showed that the average length of the wild-type petiole was about 10 cm, whereas the P8 petiole was typically 25 cm to 30 cm long (Fig. 1c). The epidermal cells of the mature petioles from P8 and Ruegen were further examined using scanning electron microscopy. We found that the P8 mutant had much longer epidermal cells than the wild type (Fig. S1, see online supplementary material), indicating that the petiole length of P8 was mainly caused by cell elongation in the longitudinal direction. Consistent with our observation, the total anthocyanin content in P8 was dramatically reduced compared to Ruegen (Fig. 1d). Similar to the petioles, the hypocotyls of P8 were also significantly elongated (Fig. 1e and f). Taken together, the most prominent phenotypes of P8 are increased tissue elongation and reduced pigmentation.

### *FvePhyB* is the primary candidate for the P8 mutation

**Figure 2:**
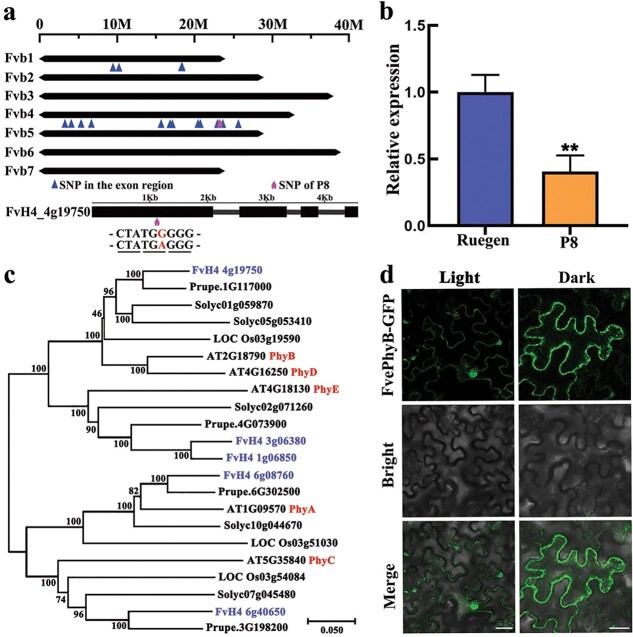
Identification and characterization of *FvePhyB.***a** Diagram showing the candidate exonic SNPs and causal gene. The red letter indicates the SNP in FvH4_4g19750 that results in premature termination of protein translation. **b** The expression level of *FvePhyB* in the petioles of Ruegen and P8, as analysed by qRT-PCR. Data are the mean ± SD of three biological replicates. ^**^P < 0.01, Student’s *t*-test. **c** Phylogenetic tree of the PHYs of Arabidopsis, *Fragaria vesca*, tomato, rice, and peach using full-length protein sequences. **d** Subcellular localization of FvePhyB-GFP in tobacco leaves. GFP fluorescence signals are shown in green. Scale bars: 25 μm.

To determine the causal gene of the P8 mutant, it was backcrossed with the wild-type Ruegen. All F_1_ plants looked similar to the wild type, indicating that P8 is a recessive mutant. The ratio of F_2_ mutant to F_2_ wild type was 9:68, much lower than 1:3 but higher than 1:15. Considering that the seed germination rate of P8 was greatly reduced (Fig. S2, see online supplementary material), we think that P8 should be caused by a single gene. Young leaves from 20 F_2_ wild type and 13 F_2_ P8 mutants were collected for whole genome resequencing. Data analysis revealed 18 candidate single nucleotide polymorphisms (SNPs) located on chromosome 1 and chromosome 4 (Fig. 2a). Among them, two SNPs caused premature termination, eight SNPs resulted in altered amino acids, and eight SNPs did not affect the protein sequence (Table S1, see online supplementary material). According to the phenotypes of P8, the red/far-red photoreceptor gene *FvePhyB* (FvH4_4g19750), whose mutation occurred in the first exon resulting in premature termination of translation, was considered the primary candidate (Fig. 2a). The SNP in *FvePhyB* was confirmed to be homozygous in 36 F_2_ mutants, while other SNPs were eliminated (Table S1, see online supplementary material). The expression level of *FvePhyB* in the P8 mutant was significantly reduced compared to the wild type, as assessed by qRT-PCR (Fig. 2b), indicating the induction of nonsense-mediated decay.

There are five phytochrome members in *Arabidopsis*, namely PhyA-E [42]. To better understand their functions, phylogenetic analysis was performed using the phytochrome homologs in *Arabidopsis, F. vesca*, *Solanum lycopersicum*, *Oryza sativa* and *Prunus persica.* In *F. vesca*, there is one FvePhyA (FvH4_6g08760), one FvePhyC (FvH4_6g40650), and two FvePhyE (FvePhyE1, FvH4_3g06380; FvePhyE2, FvH4_1g06850); FvePhyB (FvH4_4g19750) shows high similarity to both Arabidopsis PhyB and PhyD (Fig. 2c). The previous transcriptome data showed that *FvePhyA*, *FvePhyB*, and *FvePhyC* are abundantly expressed in most tissues, *FvePhyE2* is highly expressed in some tissues, whereas *FvePhyE1* is lowly expressed in all tissues (Fig. S3, see online supplementary material). Transient expression of *FvePhyB-GFP* in tobacco leaves suggests that FvePhyB is localized in the cytoplasm in the dark and can translocate to the nucleus in the light (Fig. 2d), a known feature of PhyB [43].

### *FvePhyB* overexpression causes shorter hypocotyl and delayed flowering in Arabidopsis

**Figure 3:**
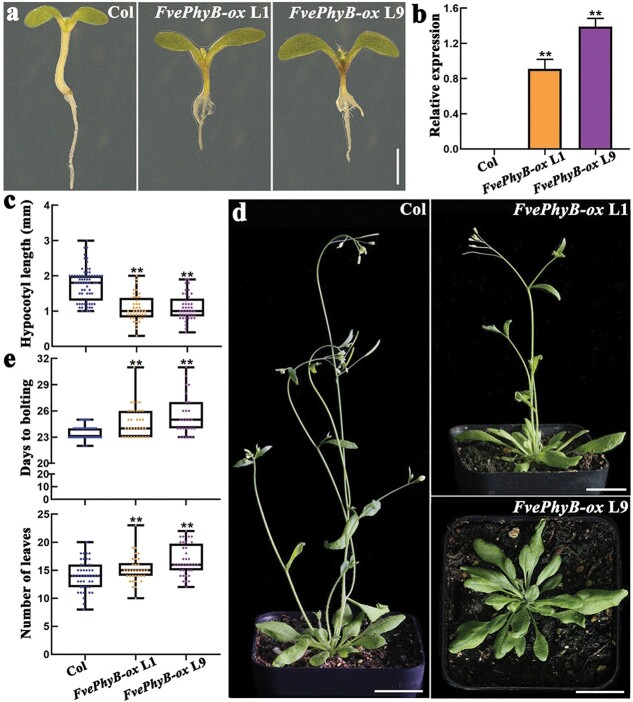
Phenotypes of the *FvePhyB-ox* transgenic lines in Arabidopsis. **a** The seedlings of wild type Arabidopsis and *FvePhyB-ox* in the T_2_ generation. **b** The expression level of *FvePhyB* in the leaves of wild-type and two *FvePhyB-ox* transgenic lines in the T_1_ generation, as analysed by qRT-PCR. Data are the mean ± SD of three biological replicates. **c** Hypocotyl length of wild type Arabidopsis and *FvePhyB-ox* (L1 and L9) in the T_2_ generation. *n* ≥ 41. **d** Plants of wild type Arabidopsis and *FvePhyB-ox* (L1 and L9) in the T_2_ generation. **e** Days to bolting and number of leaves at bolting of wild type Arabidopsis and *FvePhyB-ox* (L1 and L9) in the T_2_ generation. *n* ≥ 27. ^**^P < 0.01, Student’s *t*-test. Scale bars: 1 mm (**a**), 2 cm (**c**).

To better understand the functions of *FvePhyB*, the overexpression construct *35S::FvePhyB-GFP* was transformed into wild-type Arabidopsis Col-0. A total of 23 transgenic plants were obtained in the T_1_ generation and had similar phenotypes. Two *FvePhyB-ox* lines (L1 and L9) with significantly shorter and redder hypocotyls in the T_2_ generation were selected for detailed characterization (Fig. 3a). *FvePhyB* was abundantly expressed in both L1 and L9 examined by qRT-PCR, but the expression level in L9 was slightly higher than in L1 (Fig. 3b). In particular, the *FvePhyB-ox* hypocotyls were only about half as long as the wild type (Fig. 3c). We also found that the two *FvePhyB-ox* transgenic lines exhibited a delayed flowering phenotype under long-day conditions (Fig. 3d). Consistently, the days to bolting and the number of leaves at bolting were greater in the two *FvePhyB-ox* transgenic lines than in the wild type (Fig. 3e). In conclusion, overexpression of *FvePhyB* in Arabidopsis results in shorter hypocotyls and delayed flowering.

### *FvePhyB* promotes red light photomorphogenesis

**Figure 4:**
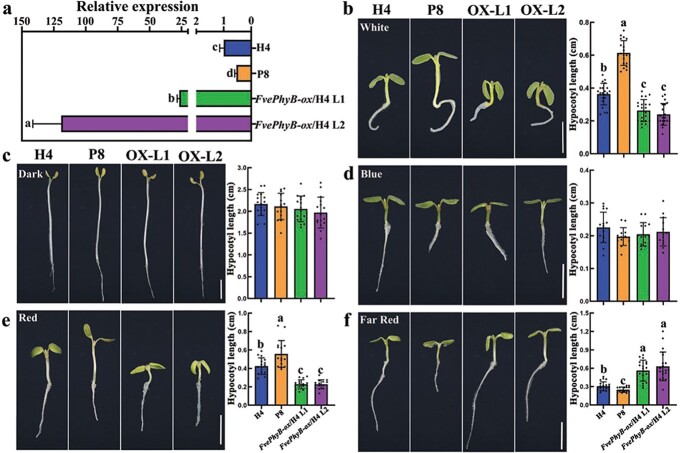
Growth of wild type, P8 and *FvePhyB-ox* seedlings under different light conditions. **a** The expression level of *FvePhyB* in the petiole of wild-type H4, P8, and two *FvePhyB-ox* transgenic lines in the T_0_ generation, as analysed by qRT-PCR. Data are the mean ± SD of three biological replicates. **b** Seedlings and hypocotyl length of wild-type H4, P8, and *FvePhyB-ox* (L1 and L2) in continuous white light, *n* ≥ 20. **c** Seedlings and hypocotyl length of wild-type H4, P8, and *FvePhyB-ox* (L1 and L2) in the dark. *n* ≥ 14. **d** Seedlings and hypocotyl length of wild-type H4, P8, and *FvePhyB-ox* (L1 and L2) in continuous blue light, *n* ≥ 14. **e** Seedlings and hypocotyl length of wild-type H4, P8, and *FvePhyB-ox* (L1 and L2) in continuous red light. *n* ≥ 14. **f** Seedlings and hypocotyl length of wild-type H4, P8, and *FvePhyB-ox* (L1 and L2) in continuous far-red light. *n* ≥ 16. Scale bars: 0.5 cm. Different letters indicate significant difference at *P* < 0.05 using Tukey’s test.

The *35S::FvePhyB-FLAG* construct was also transformed into the wild-type strawberry variety H4 due to its higher transformation efficiency. A total of eight *FvePhyB-ox* transgenic lines were obtained in the T_0_ generation. The expression of *FvePhyB* in two lines (L1 and L2) was significantly higher than that of H4 examined by qRT-PCR, with a higher level in L2 (Fig. 4a). To confirm the light response of these materials, seedlings were grown under different light conditions, including white light, dark, blue light, red light, and far red light. Consequently, P8 was taller and the *FvePhyB-ox* seedlings were shorter than the wild type under white light (Fig. 4b). However, the wild-type, P8, and *FvePhyB-ox* seedlings showed similar hypocotyl elongation in the dark and under blue light (Fig. 4c and d). The hypocotyl phenotypes of P8 and *FvePhyB-ox* under red light were similar to those under white light (Fig. 4e). In contrast, the length of P8 and *FvePhyB-ox* hypocotyls under far-red light showed a completely opposite change to that under white and red light (Fig. 4f). These results suggest that the strawberry *FvePhyB* is also a red/far-red photoreceptor during photomorphogenesis.

### Overexpression of *FvePhyB* rescues P8 mutant defects

**Figure 5:**
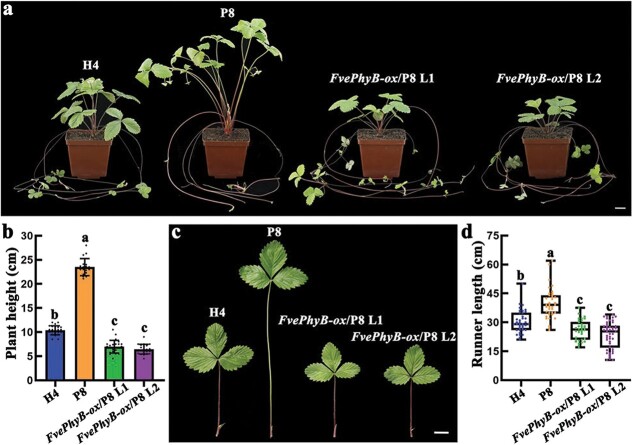
*FvePhyB-ox* rescues the P8 defects. **a** Whole plant of wild-type H4, P8, *FvePhyB-ox*; P8 (L1 and L2). **b** Plant height of wild-type H4, P8, *FvePhyB-ox*; P8 (L1 and L2), *n* ≥ 18. **c** The leaf of wild-type H4, P8, *FvePhyB-ox*; P8 (L1 and L2). **d** The length of the first internode in the runners of wild-type H4, P8, *FvePhyB-ox*; P8 (L1 and L2), *n* ≥ 34. Different letters indicate significant difference at *P* < 0.05 using Tukey’s test. Scale bars: 2 cm.

To confirm that *FvePhyB* is the causal gene for P8, the two *FvePhyB-ox* transgenic lines in H4 were crossed with the P8 mutant. *FvePhyB* overexpression lines with the homozygous P8 mutation were obtained in the F_2_ generation. Phenotypic observations showed that *FvePhyB-ox* (L1 and L2) could dramatically reduce the plant height of the P8 mutant, which is even shorter than the wild type (Fig. 5a, b). In addition to the petiole length, the petiole color of *FvePhyB-ox*/P8 became as red as that of H4 (Fig. 5c). Consistent with our observation, the total anthocyanin content was restored to the wild-type level in two *FvePhyB-ox*/P8 lines (Fig. S4, see online supplementary material). We also found that the length of the first internode in the runners of P8 was significantly longer than that of H4 (Fig. 5d). The increased elongation of the runners in P8 was due to cell elongation in the longitudinal direction according to the SEM images of the epidermal cells (Fig. S5, see online supplementary material). In contrast, the length of the first internode in the *FvePhyB-ox*/P8 runners was significantly shorter than that in H4 (Fig. 5d). Therefore, we conclude that *FvePhyB* is indeed the causal gene for P8 according to this complementation assay.

### Differentially expressed genes in the petioles and fruit receptacle of P8

**Figure 6:**
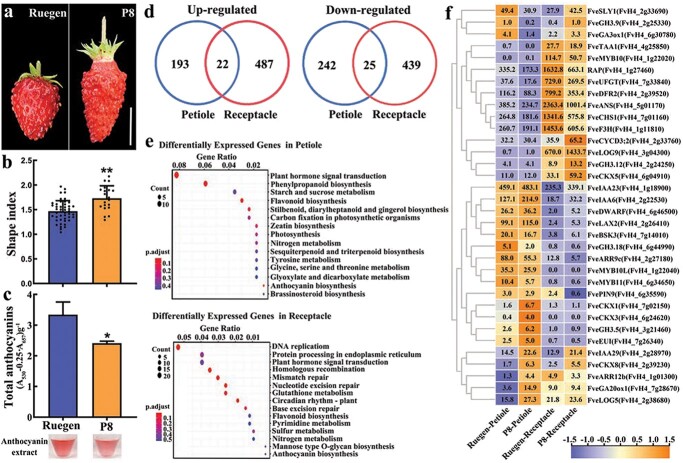
Transcriptome analysis of young petioles and fruit receptacles at the turning stage of P8. **a** Mature fruits of wild-type Ruegen and P8. Scale bar: 1 cm. **b** The shape index of mature fruits of wild-type Ruegen and P8, *n* ≥ 20. **c** Total anthocyanin contents of mature fruits of wild-type Ruegen and P8. The upper image shows the anthocyanin extract. Data are the mean ± SD of three biological replicates. ^*^P < 0.05; ^**^P < 0.01; Student’s *t*-test. **d** Venn diagram shows the specific and common genes of up-regulated and down-regulated (fold change >1.5, padj <0.05) in young petioles and fruit receptacles. **e** Top 15 enriched KEGG pathways among the DEGs in young petioles and fruit receptacles. The X-axis indicates the enrichment factor. The dot size indicates gene number. The dot color indicates the adjusted *P* value. **f** Heatmap showing the expression levels of the DEGs in the hormone and anthocyanin pathways. The TPM values are shown in the heatmap. Color scale indicates log_2_(TPM + 1).

In addition to the vegetative organs, the fruits of P8 were longer in shape and lighter in color than the fruits of the wild type Ruegen (Fig. 6a). Consistently, the fruit shape index of P8 was slightly higher than wild type (Fig. 6b), and the total anthocyanin content was significantly lower in mature fruits of P8 (Fig. 6c). To investigate the underlying molecular basis of the P8 defects in petiole and fruit, the petioles with folded green leaves and fruit receptacles (without achenes) at the turning stage were collected from P8 and Ruegen for RNA-seq. Each sample had three biological replicates, resulting in a total of 12 libraries. More than 34 million reads were obtained for each library. On average, 90.83% of the raw reads could be uniquely mapped against the *F. vesca* reference genome ver4.0 [44] (Table S2, see online supplementary material). Differentially expressed genes (DEGs) were identified in petioles and receptacles of P8 compared to those of Ruegen (Data S1, see online supplementary material, fold change >1.5, padj <0.05). As a result, 215 genes were up-regulated and 267 genes were down-regulated in P8 petioles, 509 genes were up-regulated and 464 genes were down-regulated in P8 receptacles (Fig. 6d). There are very few common up- or down-regulated genes between petioles and receptacles, suggesting a quite different transcriptome profile in these tissues. The top 15 enriched KEGG pathways were identified for the DEGs in petioles and fruit receptacles, including plant hormone signal transduction, flavonoid biosynthesis, etc. (Fig. 6e).

Among the DEGs, expression levels of 25 genes in the auxin, gibberellin (GA), cytokinin, and brassinolide pathways and eight genes in the anthocyanin pathway were shown in the heatmap (Fig. 6f). Some of the hormone genes were upregulated in both tissues at P8, such as *FveIAA6* (*Indole-3-Acetic Acid 6*), *FveIAA29*, and *FveCKX8* (*cytokinin oxidase 8*), while *FveARR9c* was downregulated in both tissues (Fig. 6f). Some genes were up-regulated in only one tissue, such as *FveGH3.5* (*Gretchen Hagen 3.*5), *FveGA20ox*1 (*Gibberellin 20-oxidase 1*), *FveEUI* (*Elongated Uppermost Internode*), *FveARR12b*, *FveLOG5* (*Lonely Guy 5*), *FveCKX1*, and *FveCKX3* in the petioles, *FveIAA23*, *FveGH3.12*, *FveLAX2*, *FveLOG9*, *FveCKX5*, *FveCYCD3;2*, *FveBSK3* (*Brassinosteroid-Signaling Kinase*)*,* and *FveDWARF* in fruit receptacles. In the anthocyanin pathway, most structural genes (*FveCHS1*, *FveF3H*, *FveDFR2*, *FveANS*, and *FveUFGT*) and the transporter gene *RAP* were downregulated in both tissues. In contrast, *FveMYB10* was specifically reduced in P8 fruit receptacles, whereas *FveMYB10L* and *FveMYB11* (FvH4_6g34650) were specifically reduced in P8 petioles (Fig. 6f). Expression levels of other reported regulators in the anthocyanin pathway were not significantly changed in this data. To confirm these results, the expression levels of five hormone genes and seven anthocyanin genes were examined by qRT-PCR in the same tissues. These genes showed the same expression patterns as the RNA-seq data (Fig. S6, see online supplementary material). In conclusion, *FvePhyB* can alter the length and color of petioles and fruits by regulating the expression levels of some well-known hormone and anthocyanin biosynthesis genes.

### *FvePhyB* and *FveRGA1* synergistically regulate internode length

**Figure 7:**
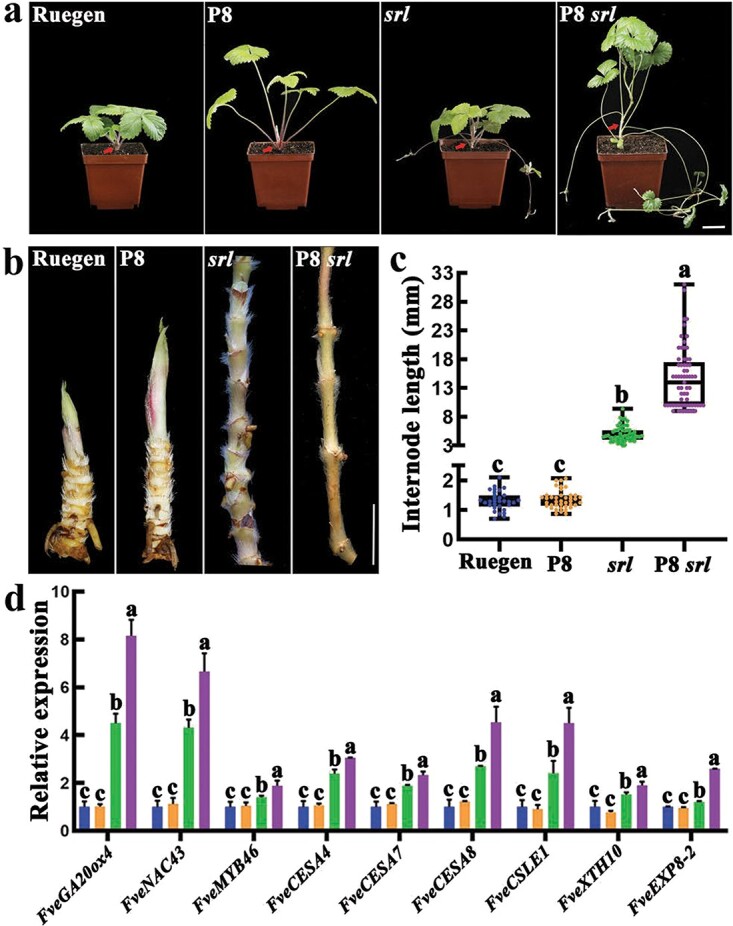
Characterization of the P8 *srl* double mutant in *Fragaria vesca.***a** Whole plant of wild-type Ruegen, P8, *srl*, and P8 *srl*. The red arrows indicate the internodes. **b** The stems with leaves removed of wild-type Ruegen, P8, *srl*, and P8 *srl*. **c** The average length of the internodes of wild-type Ruegen, P8, *srl*, and P8 *srl*, n ≥ 31. **d** The expression level of some selected genes in the stems of wild-type Ruegen, P8, *srl*, and P8 *srl*. Data are the mean ± SD of three biological replicates. Different letters indicate significant difference at *P* < 0.05 using Tukey’s test. Scale bars: 3 cm (**a**), 1 cm (**b**).

Light signaling and the GA pathway have opposing effects on hypocotyl elongation [45]. Transcriptome data showed that *FvePhyB* may also interact with the GA pathway in *F. vesca* (Fig. 6f). The *srl* mutant in *F. vesca* was caused by a mutation in *FveRGA1*, which encodes a DELLA protein in GA signaling [46]. To test the genetic interaction between *FvePhyB* and *FveRGA1*, the P8 *srl* double mutant was generated, which has longer stems than the P8 or *srl* single mutant (Fig. 7a). When all the leaves were removed, it was clear that the internode of *P8 srl* was the longest among these mutants (Fig. 7b). Measurements showed that the P8 internode was comparable to the wild-type internode, while the introduction of P8 into *srl* could dramatically increase internode elongation (Fig. 7c). Furthermore, the cortex cells of the stem internodes were examined in these mutants. The cell length in P8 remains the same as in WT, the cell length in *srl* is slightly increased, while the cell length in P8 *srl* is greatly increased, suggesting that cell elongation mainly contributes to the internode elongation (Fig. S7, see online supplementary material). These results indicate that *FvePhyB* and *FveRGA1* together regulate the internode length.

To investigate the genes involved in the internode elongation, the developing internodes of wild type, P8, *srl*, and P8 *srl* were collected for qRT-PCR analysis. The expression of the GA biosynthesis gene *FveGA20ox4* was not significantly different between P8 and Ruegen, while it was more highly expressed in *srl* and most abundant in P8 *srl* (Fig. 7d). The expression levels of the cell wall regulatory transcription factors *FveNAC43*, *FveMYB46*, the cell wall synthesis and metabolism genes *FveCESA4* (*cellulose synthase 4*), *FveCESA7*, *FveCESA8*, *FveCSLE1*, *FveXTH10* (*xyloglucan endotransglucosylase/Hydrolase 10*) and the cell wall expansion gene *FveEXP8–2* (*Expansin8–2*) showed the same trend as that of *FveGA20ox4* (Fig. 7d). These results suggest that *FvePhyB* and *FveRGA1* may interact to modulate the cell wall synthesis and expansion in the process of internode elongation.

## Discussion

Strawberries are often grown under shelter throughout the world, which results in reduced light intensity and altered light quality, leading to plant overgrowth. Plants have evolved multiple photoreceptors and a complex signaling network to adapt to changes in light. Phytochromes are red/far-red light receptors that regulate photomorphogenesis, flowering time, shade avoidance, stomatal development, and axillary bud outgrowth [13, 47–49]. In addition, phytochrome B has also been implicated as a thermosensor [9, 50], another important environmental factor for plant development. However, no functional analysis has been described for the phytochromes in strawberry. In this study, we identified the *PhyB* mutant P8 in *F. vesca*, which allows us to systematically assess the functions of light signaling in strawberry plant development.

### FvePhyB is a red/far-red light receptor

Phylogenetic analysis showed that the five PHYs in Arabidopsis are divided into the PHYA/C and PHYB/D/E clades. In *F. vesca*, there is one FvephyA, one FvePhyB (homologous to Arabidopsis PhyB and PhyD), one FvePhyC, and two FvePhyEs (FvePhyE1 and FvePhyE2) (Fig. 2). Transcriptome data showed that *FvePhyA*/*B*/*C*/*E2* are abundantly expressed (Fig. S3, see online supplementary material), suggesting that they may have important functions. However, *FvePhyE1* is very lowly expressed and thus may not be functional. Genetic studies revealed that *FvePhyB* is the causal gene for the P8 mutant in *F. vesca*, which is the first reported mutant of the phytochrome genes in strawberry. The translocation of FvePhyB from the cytoplasm to the nucleus in response to light treatment is similar to that of Arabidopsis PhyB [43, 51]. Overexpression of *FvePhyB* in Arabidopsis caused shorter hypocotyls and late flowering (Fig. 3). Furthermore, the P8 mutant and *FvePhyB-ox* transgenic strawberry lines show the defects specifically under red and far-red light (Fig. 4). These results suggest that FvePhyB is an ortholog of PhyB with conserved functions in red/far-red light sensing.

### *FvePhyB* promotes tissue pigmentation by regulating the anthocyanin pathway

The effect of different light qualities on strawberry fruit coloration has been tested and blue light has been shown to be the most efficient in enhancing pigmentation [52, 53]. As these studies used only one light intensity for each monochromatic light, this conclusion is controversial. The strongly reduced anthocyanin content at P8 suggests that red light also plays an essential role in pigmentation. Compared to dark, white light treatment stimulates anthocyanin accumulation in strawberry fruits by regulating *MYB10* at both transcriptional and post-transcriptional levels [54, 55]. Consistent with this result, *FvMYB10* and several other anthocyanin biosynthetic genes are significantly down-regulated in P8 fruits (Fig. 6). Different from fruits, *FveMYB10L* is highly expressed and acts as a key regulator of pigmentation in petioles [36]. As expected, *FveMYB10L* expression is significantly reduced in P8 petioles. These results demonstrate that *FvePhyB* mediates light-controlled pigmentation via different transcription factors.

The signaling network of *FvePhyB* in anthocyanin regulation remains elusive. Typically, PhyB can inhibit the activities of PIFs and the COP1/SPA complex in the nucleus [56]. Several PIFs have been reported to suppress pigmentation in Arabidopsis and apple [28, 57]. The COP1/SPA complex is responsible for the degradation of HY5. White light treatment of strawberry fruits induces the expression of *FveHY5* and *FvebHLH9*, whose products interact together and activate the expression of the anthocyanin structural gene *FveDFR* [29]. Stable overexpression of *FveHY5* (fused to VP16) or *FaBBX22* (*B-box domain protein 22*) in strawberry greatly enhanced pigmentation in various tissues [53]. In other plant species, HY5 can interact with BBX transcription factors to regulate anthocyanin accumulation [58–61]. In our transcriptome data, the expression level of *FveHY5* is not significantly altered at P8, probably because both P8 and wild-type plants are grown under a constant 16 h light/8 h dark photoperiod. It is possible that FveHY5 protein levels are altered in P8 fruits. Further studies are needed to prove whether the PIFs and the COP1-HY5 module are involved in *FvePhyB*-mediated coloration.

### *FvePhyB* regulates tissue elongation through hormone pathways

The P8 mutant has elongated hypocotyls, petioles and runners, which are similar to *phyB* mutants in other plant species. To find the genes affected by *FvePhyB*, the developing petioles of P8 and wild type were subject to transcriptome analysis. Phytochrome B is known to interact with various hormones by influencing their biosynthesis and signaling pathways during tissue elongation [62]. Consistently, the plant hormone signal transduction pathway is enriched in the DEGs in P8 petioles (Fig. 6; Fig.S6, see online supplementary material). In the downstream of the hormone signaling pathway, the cell wall synthesis and metabolism genes and cell wall expansion genes are transcriptionally regulated.

Another interesting phenotype of P8 is that the ripe fruit has a higher shape index (length/width ratio). The fruit shape in strawberry is largely regulated by auxin and GA [63]. In particular, excess auxin leads to rounder fruits, while excess GA leads to longer fruits. Therefore, the fruit shape of P8 is probably related to the auxin or GA pathway. Mutants of the auxin signaling gene *FveARF8* in *F. vesca* are known to have rounder fruits [64]. The study in Arabidopsis showed that PhyB directly interacts with ARF8 and ARF6 [14], suggesting a possible mechanism for *FvePhyB*-regulated fruit shape.

Similar to Arabidopsis, strawberry has a rosette growth habit. The Arabidopsis *phya phyb phye* triple mutant, but not the single or double mutants, has a significantly longer internode than the wild type [65], suggesting a functional redundancy of these phytochromes in inhibiting internode elongation. The internode length of P8 remains the same as in the wild type, indicating that a single mutation of *FvePhyB* is not sufficient to promote internode elongation. The *srl* mutant of the DELLA gene *FveRGA1* in GA signaling has elongated internodes, whereas the mutation of all five DELLA genes in Arabidopsis had no effect on internode length [66], indicating less functional redundancy of strawberry DELLAs. Internode elongation in *srl* was dramatically enhanced by P8 (Fig. 7), suggesting functional redundancy between *FvePhyB* and *FveRGA1*. The PIF homologs may be involved in the synergistic interaction of *FvePhyB* and *FveRGA1*, as suggested by the Arabidopsis study showing a physical interaction between PIF4 and RGA [16]. In rice, the DELLA protein SLR1 can directly interact with the transcription factors NAC29/31 to inhibit the expression of downstream MYB61 and cellulose synthase genes [67]. In *Arabidopsis*, SND1 (secondary wall-associated NAC domain 1) and NST1 (NAC secondary wall thickening promoting factor1) could directly regulate the transcription levels of *ATMYB46* and *ATMYB83* by binding the SNBE (secondary wall NAC binding element) element, and thereby regulating the cellulose synthesis and degradation [68–70]. Accordingly, the up-regulated *FveNAC43*, most similar to *NAC29* in rice and *NST1* in Arabidopsis, and *FveMYB46*, most similar to *ATMYB46*, could be the upstream regulators of the cellulose synthase genes (Fig. 7). Nevertheless, the simultaneous knockout of *FvePhyB* and *FveRGA1* resulted in much longer stems, a dramatic change in plant architecture.

## Materials and methods

### Plant materials and growth conditions

Two *F. vesca* varieties (Hawaii 4 and Ruegen) were used in this study. The P8 mutant was obtained from Ruegen by EMS mutagenesis. Plants were grown under the following conditions: light intensity, 100 μmol m^−2^ s^−1^; photoperiod, 16 h light/8 h dark; 24°C.

### Gene isolation of the P8 mutant

The causative gene of the P8 mutant was identified as previously described [40]. Briefly, the M_2_ P8 mutant was backcrossed to the parent Ruegen. The same amounts of young leaves from 13 F_2_ mutant and 20 F_2_ wild type plants were pooled for genomic DNA extraction using a CTAB method. Each group had ~6G of raw reads (150 bp, paired-end) using the Illumina HiSeq X Ten platform (Biomarker Technologies, Beijing, China). Candidate SNPs were identified using the established pipeline [40] and further confirmed by PCR amplicon sequencing in additional F_2_ P8 mutants (Table S1, see online supplementary material).

### Scanning electron microscopy

Approximately 0.5 cm of petioles or runners were collected and immediately placed in 2.5% glutaraldehyde for fixation overnight at 4°C, washed with phosphate-buffered saline (PBS, 0.1 M, without NaCl) 3–5 times (15 min each time), dehydrated with a series of alcohols (30%, 50%, 70%, 80%, 90%, 95%, 100%) successively for 15 min each time, then thoroughly dehydrated with 100% alcohol 1–2 times, transferred to isoamyl acetate twice (20 min each time), critical point dried, vacuumed and coated with gold for 45 s, and photographed by scanning electron microscope (JSM-6390LV).

### Plasmid construction

For *35S::PhyB-GFP*, the coding sequence without the stop codon was cloned directly into pRI101 at the *Nde*I and *EcoR*I sites. For *35S::PhyB-FLAG*, the coding sequence without the stop codon was inserted into pENTR1A at the *Sal*I and *Kpn*I sites to fuse with FLAG, and then combined into pK7WG2D by the gateway cloning method. The primers are shown in Table S3 (see online supplementary material).

### Phylogenetic analysis

The PhyB proteins were downloaded from NCBI (www.ncbi.nlm.nih.gov), TAIR (www.arabidopsis.org), and PLAZA (bioinformatics.psb.ugent.be/plaza). The phylogenetic tree was constructed in MEGA7. The statistical methods of neighbor-joining and bootstrap analysis (1000 replicates) were used.

### Expression pattern analysis

The TPM (transcripts per kilobase of exon model per million mapped reads) values of the phytochrome genes in different tissues were obtained from the published transcriptome data [71]. A heatmap showing their expression patterns was generated using TBtools software.

### Subcellular localization analysis

*Agrobacterium* colonies of 35S::*FvePhyB-GFP* were grown overnight at 28°C in 2 ml liquid LB medium with 50 mg L^−1^ kanamycin. The culture was spun down and resuspended in buffer (5 g L^−1^ D-glucose, 50 mM MES, 2 mM Na_3_PO_4_, 100 μM acetosyringone, pH 5.6) to OD_600_ of 0.4. The suspension was infiltrated into 3-week-old tobacco (*Nicotiana benthamiana*) leaves by syringe in the dark. Some tobacco leaves were covered with tin foil for dark treatment, while others were grown under normal light conditions. Images of GFP fluorescence were taken after 48 hours using a confocal microscope (TCS SP8; Leica Wetzlar, Germany).

### Arabidopsis transformation

Transformation of *35S::PhyB-GFP* into Arabidopsis Col-0 was carried out using the floral-dip method [72]. The T_1_ transgenic seeds were screened on half-strength MS (M5524, Sigma-Aldrich) containing 100 mg L^−1^ kanamycin.

### Strawberry transformation

*Agrobacterium*-mediated transformation of the wild strawberry *F. vesca* was described previously [73]. The positive transformants were screened with both kanamycin and GFP fluorescence using a fluorescence dissecting microscope (Micro-shot Technology Limited, Guangzhou, China, MZX81). Carbenicillin and timentin (both 250 mg L^−1^) were applied to inhibit *Agrobacterium* growth.

### RNA-seq and data analysis

Total RNA was extracted from young petioles before the leaves were unfolded and fruit receptacle at turning stage of P8 and Ruegen using the HiPure Plant RNA Mini Kit (Magen, Guangzhou, China, R4151–02). Three biological replicates were tested for each sample. RNA-seq libraries were constructed using the PE150 method and sequenced on the Illumina HiSeq X Ten platform (Personalbio, Shanghai, China). Raw reads from each library (~8G) were aligned to the *F. vesca* reference genome using the STAR program in 2-pass mode [71, 74]. The reads for each gene were counted using FeatureCounts [75]. Differentially expressed genes were identified by using the R package DESeq2 (fold change >1.5, *Padj* <0.05) [76].

### Quantitative RT-PCR

Total RNA was extracted using a HiPure Plant RNA Mini Kit (Magen, Guangzhou, China, R4151–02). Approximately 1 μg of total RNA was used for cDNA synthesis using a PrimeScript™ RT Reagent Kit (TaKaRa, Shiga, Japan, Cat# RR047A). qRT-PCR procedures were performed as previously described [41]. FvH4_4g24420 in *F. vesca* and AT3G18780 in Arabidopsis were used as the internal controls. The 2^−∆∆CT^ method was used to obtain gene expression levels [77]. Primers are listed in Table S3 (see online supplementary material).

### Measurement of total anthocyanins

About 0.5 g of fresh strawberry fruits or petioles were ground into powder in liquid nitrogen, added to 5 ml of extraction solution (methanol: H_2_O: formic acid: trifluoroacetic acid = 70:27:2:1) and kept in the dark for 12 h at 4°C. After filtration, the supernatant absorbance was determined at 530 nm and 657 nm using an ultraviolet spectrophotometer (Hoefer Vision, SP-2001). Total anthocyanin content was obtained using Qanthocyanins = [A_530_-(0.25 × A_657_)]/M, where A_530_ and A_657_ refer to the absorbance at the 530 nm and 637 nm wavelengths, and M indicates the fresh weight of the sample [41]. All samples had three independent biological replicates.

### Seedling growth under different light conditions

After 2 weeks of storage at 4°C, the seeds of wild-type H4, the P8 mutant and two independent *FvePhyB-ox* transgenic lines were sterilized and placed on Petri dishes containing 1/2 MS medium (2.22 g L^−1^ MS, 20 g L^−1^ sucrose, 7 g L^−1^ agar, pH 5.8). After 2 days of cultivation under white light (100 μmol m^−2^ s^−1^, 16/8 h light/dark), the seeds were then germinated and grown under continuous dark, white light, red light (65 μmol m^−2^ s^−1^), far red light (13 μmol m^−2^ s^−1^), and blue light (32 μmol m^−2^ s^−1^) at 24°C. Hypocotyl length was measured when the cotyledons were fully expanded.

### Statistical analyses

The program SPSS v22.0 (IBM Corp., Armonk, NY, USA) was used for statistical analyses. Student’s *t*-test was used for pairwise comparisons, and Tukey’s test was used for multiple-sample comparisons.

## Supplementary Material

Web_Material_uhad232

## Data Availability

All relevant data can be found within the manuscript and its supplementary materials. The transcriptome reads have been submitted to the Sequence Read Archive at NCBI. The accession number is PRJNA997061.
