# Supplementary material for: The red/far-red light photoreceptor FvePhyB regulates tissue elongation and anthocyanin accumulation in woodland strawberry
Source: Hortic Res. 2023 Nov 17;10(12):uhad232. doi: 10.1093/hr/uhad232 (PMC10745270; doi:10.1093/hr/uhad232)
Supplement: Web_Material_uhad232 [file web_material_uhad232.zip › Supplemental figures and tables-revised.docx]

**The red/far-red light photoreceptor FvePhyB regulates tissue elongation and anthocyanin accumulation in woodland strawberry**

**Running title: Roles of *FvePhyB* in strawberry**

Qi Gao^1,2^, Shaoqiang Hu^1,2^, Xiaoli Wang^1,2^, Fu Han^1,2^, Huifeng Luo^3^, Zhongchi Liu^4^ and Chunying Kang^1,2,*^

1. National Key Laboratory for Germplasm Innovation & Utilization of Horticultural Crops, Huazhong Agricultural University, Wuhan, 430070, China

2. Hubei Hongshan Laboratory, Wuhan, 430070, China

3. Institute of Horticulture, Hangzhou Academy of Agricultural Sciences, Hangzhou, 310024, China

4. Department of Cell Biology and Molecular Genetics, University of Maryland, College Park, MD, 20742, USA

**Author email:**

Qi Gao, gaoqi@mail.hzau.edu.cn

Shaoqiang Hu, 870594116@webmail.hzau.edu.cn

Xiaoli Wang, Wxl2022@webmail.hzau.edu.cn

Fu Han, hanfu@webmail.hzau.edu.cn

Huifeng Luo, 949321876@qq.com

Zhongchi Liu, zliu@umd.edu

***Authors for correspondence**:

Chunying Kang

National Key Laboratory for Germplasm Innovation & Utilization of Horticultural Crops, Huazhong Agricultural University, Wuhan, 430070, China

E-mail: ckang@mail.hzau.edu.cn

Tel: +86-13871595425

Fax: +86-027-87282010

ORCID ID: 0000-0001-9269-5185


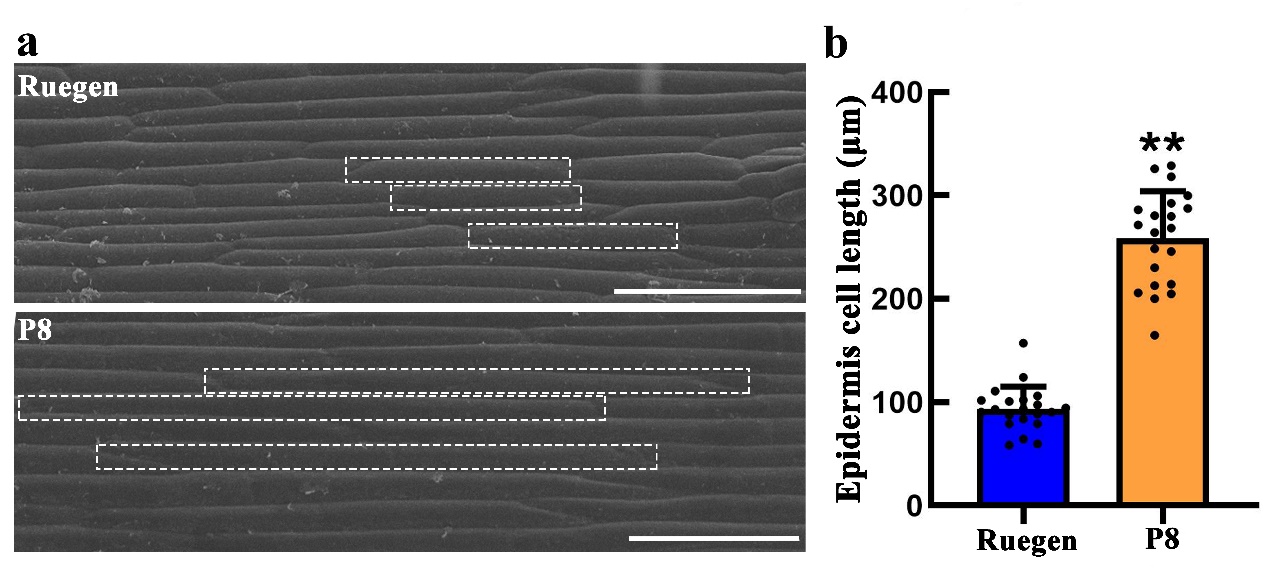


**Fig. S1 The epidermal cells in the petioles of wild-type Ruegen and P8*.***

(a) The shape of the epidermal cells in the petioles of Ruegen and P8. Scale bars: 100 μm. (b) The length of the epidermal cells in the petioles of Ruegen and P8. Data are the mean ± SD of 21 randomly selected epidermal cells. **, *P* < 0.01, Student’s *t*-test.


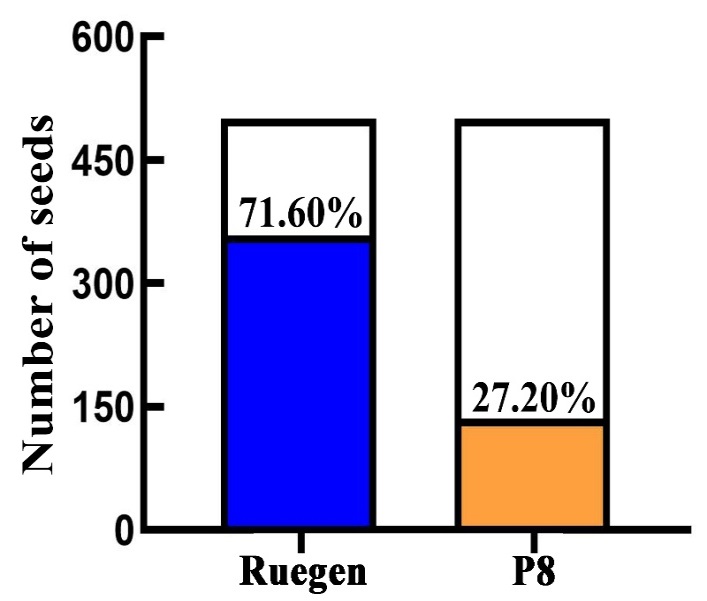


**Fig. S2 Germination rate of wild-type Ruegen and P8 seeds.**

The percentage indicates the ratio of germinated seeds to all 500 seeds.


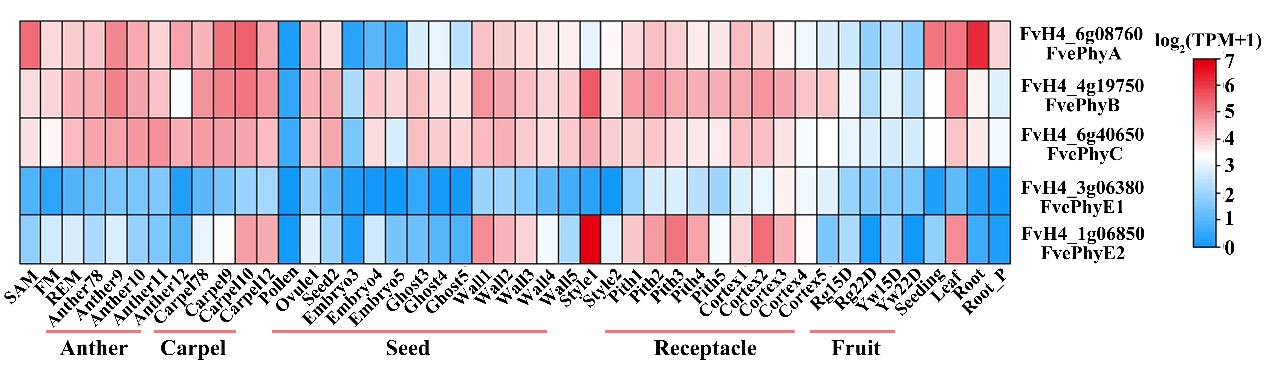


**Fig. S3** **Expression patterns of the phytochrome genes in *Fragaria vesca*.**

Heatmap showing the expression levels of the 5 phytochrome genes in *F. vesca* according to the RNA-seq data. The color scale indicates log_2_(TPM+1). Each row corresponds to a gene, and each column corresponds to a specific tissue.


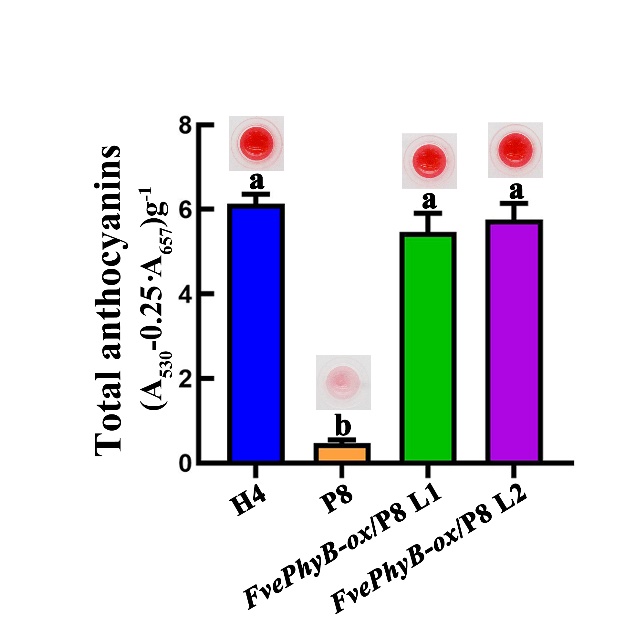


**Fig. S4 Total anthocyanin contents in *FvePhyB-ox*/P8.**

The upper images show the anthocyanin extract. Significant differences at the *P* < 0.05 level are indicated by different letters, tested by Tukey’s test.


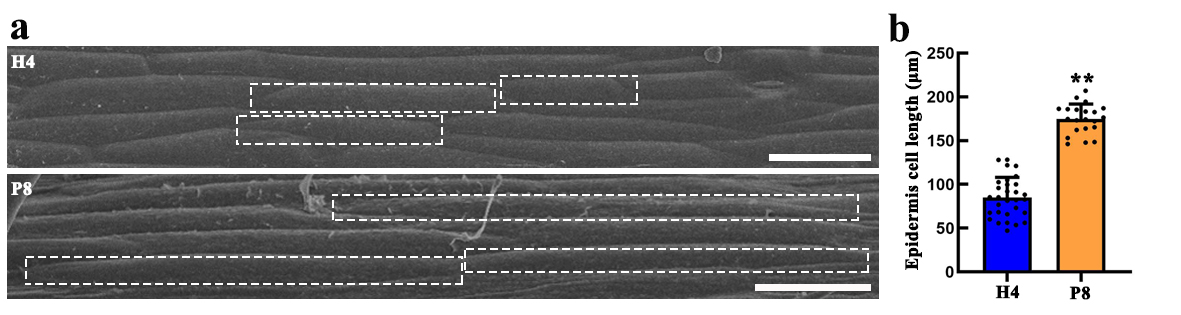


**Fig. S5 The epidermal cells in the runners of wild-type H4 and P8.**

(a) The shape of epidermal cells in the runners of wild-type H4 and P8. The middle part of the first internode in the runners was used for this observation. Scale bars: 50 μm. (b) The length of the epidermal cells in the runners of wild-type H4 and P8. Data are the mean ± SD of more than 21 randomly selected epidermal cells. **, *P* < 0.01, Student’s *t*-test.


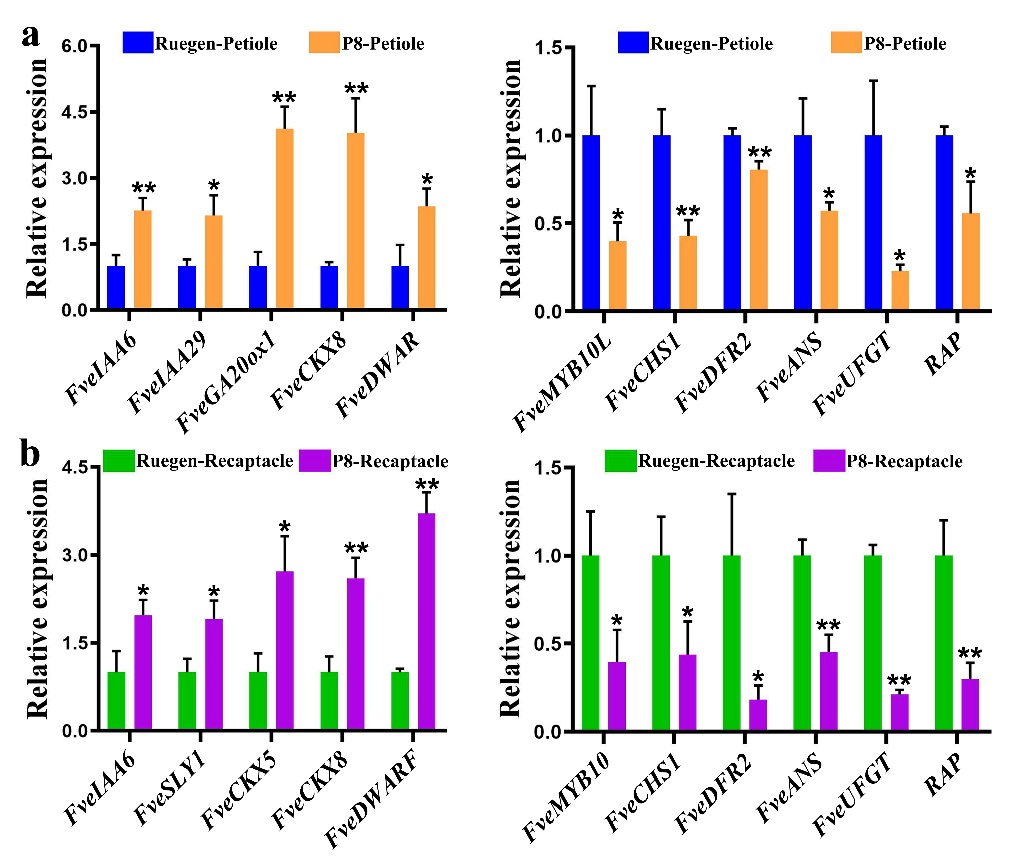


Fig. S6 Expression levels of the selected genes in the hormone and anthocyanin pathways.

(a) Expression levels of the selected genes in the young petioles of wild-type H4 and P8 examined by qRT-PCR. (b) Expression levels of the selected genes in fruit receptacles of wild-type H4 and P8 examined by qRT-PCR. Data are the mean ±SD of three biological replicates. *, *P* < 0.05; **, *P* < 0.01; Student’s *t*-test.


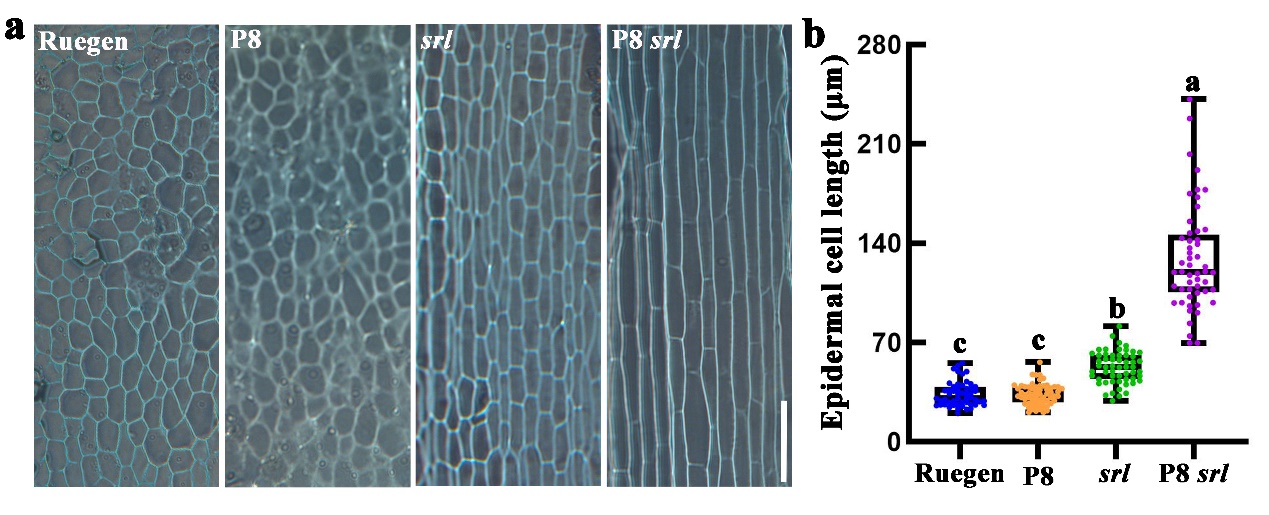


Fig. S7 Stem cell morphology in the internodes of the P8 and *srl* single and double mutants.

(a) The shape of the stem cells in the internodes of Ruegen, P8, *srl* and P8 *srl*. The third internode under the apex meristem was used for this observation. Scale bars: 100 μm. (b) The length of the stem cells in the internodes of Ruegen, P8, *srl* and P8 *srl*. Data are the mean ± SD of more than 50 randomly selected epidermal cells. Significant differences at the *P* < 0.05 level are indicated by different letters, tested by Tukey’s test.

Table S1. Candidate SNPs in the exons for P8 and validation.

| **Chromosome** | **Location** | **Base change** | **Amino acid change** | **Base ratio in mutant** | **Base ratio in wild type** | **GeneID** | **Heterozygous mutants / All examined mutants** |
| --- | --- | --- | --- | --- | --- | --- | --- |
| Fvb1 | 9,568,490 | GTT-ATT | V-F | A:93%;G:7% | A:36%;G:64% | FvH4_1g16610 | 1/8 |
| Fvb1 | 10,343,736 | TCG-TTG | S-L | T:96%;C:4% | T:47%; C:53% | FvH4_1g17790 | 1/8 |
| Fvb1 | 18,473,174 | GGC-GGT | G-G | T:96%;C:4% | T:37%; C:63% | FvH4_1g26640 |  |
| Fvb1 | 18,473,191 | GCG-GTG | A-V | T:96%;C:4% | T:38%; C:62% | FvH4_1g26640 | 1/8 |
| Fvb4 | 3,371,244 | GTT-ATT | V-I | A:100% | A:13%;G:87% | FvH4_4g03820 | 1/8 |
| Fvb4 | 4,186,775 | GTT-ATT | V-I | A:98%;G:2% | A:30%;G:70% | FvH4_4g04800 | 1/8 |
| Fvb4 | 5,473,779 | TGG-TGA | W-stop | A:98%;G:2% | A:31%;G:69% | FvH4_4g06060 | 1/6 |
| Fvb4 | 6,786,306 | GGA-GAA | G-E | A:96%;G:4% | A:26%;G:74% | FvH4_4g07420 | 1/6 |
| Fvb4 | 15,784,987 | TGC-TGT | C-C | T:98%;C:2% | T:23%; C:77% | FvH4_4g12100 |  |
| Fvb4 | 16,946,389 | AAG-AAA | K-K | A:96%;G:4% | A:29%;G:71% | FvH4_4g13340 |  |
| Fvb4 | 17,246,398 | AAG-AAA | K-K | A:100% | A:14%;G:86% | FvH4_4g13720 |  |
| Fvb4 | 20,533,042 | GAT-AAT | E-N | A:100% | A:26%;G:74% | FvH4_4g16660 | 1/8 |
| Fvb4 | 20,839,275 | ACT-ATT | T-I | T:98%;C:2% | T:20%;C:80% | FvH4_4g16900 | 1/6 |
| Fvb4 | 23,065,176 | GCA-ACA | A-T | A:100% | A:31%;G:69% | FvH4_4g19400 | 1/6 |
| Fvb4 | 23,266,400 | GCC-GCT | A-A | T:98%;C:2% | T:16%; C:84% | FvH4_4g19720 |  |
| **Fvb4** | **23,294,693** | **TGG-TGA** | **W-stop** | **A:98%;G:2%** | A:21%;G:79% | **FvH4_4g19750** | **0/36** |
| Fvb4 | 23,739,227 | AGT-AAT | S-N | A:100% | A:10%;G:90% | FvH4_4g20560 | 1/8 |
| Fvb4 | 25,749,498 | AAG-AAA | K-K | A:100% | A:17%;G:83% | FvH4_4g23180 |  |

Table S2. Summary of the RNA-seq read statistics.

| **Sample ID** | **Number of raw reads** | **Number of uniquely mapped reads** | **% mapped** |
| --- | --- | --- | --- |
| Ruegen-Petiole-1 | 42,220,096 | 38,561,530 | 91.33% |
| Ruegen-Petiole-2 | 39,257,776 | 36,068,592 | 91.88% |
| Ruegen-Petiole-2 | 39,929,240 | 36,726,004 | 91.98% |
| P8-Petiole-1 | 46,141,034 | 42,612,776 | 92.35% |
| P8-Petiole-2 | 37,220,020 | 34,391,914 | 92.40% |
| P8-Petiole-3 | 34,548,978 | 31,966,928 | 92.53% |
| Ruegen-Receptacle-1 | 44,992,096 | 40,841,952 | 90.78% |
| Ruegen-Receptacle-2 | 41,318,458 | 37,271,412 | 90.21% |
| Ruegen-Receptacle-3 | 39,405,014 | 35,580,218 | 90.29% |
| P8-Receptacle-1 | 44,860,114 | 40,133,720 | 89.46% |
| P8-Receptacle-2 | 38,603,194 | 33,820,352 | 87.61% |
| P8-Receptacle-3 | 46,207,528 | 41,205,896 | 89.18% |
| Average |  |  | 90.83% |

**Table S3. Primers used in this study.**

| **Gene ID** | **Primer name** | **Primer sequence (5'-3')** |
| --- | --- | --- |
| **Primers used for making constructs** | | |
| FvH4_4g19750 | FvePhyB-GFP-F | TCTTCACTGTTGATACATATGATGGCTTCAGCAAGCGGCAAGG |
|  | FvePhyB-GFP-R | GCCCTTGCTCACCATGAATTCCCTAGTCAACACTCTTTTCAGTATC |
| FvH4_4g19750 | FvePhyB-FLAG-F | AAAGGAACCAATTCAGTCGACATGGCTTCAGCAAGCGGCAAGG |
|  | FvePhyB-FLAG -R | TGGAAAAGGGAATTCGGTACCCCTAGTCAACACTCTTTTCAGTATC |
| **Primers used for qRT-PCR** | | |
| FvH4_4g19750 | FvePhyB-qRT-F | ATAGGATTGATGTTGGGGTTGT |
|  | FvePhyB-qRT-R | AGCAGCTTAATATCCCCACCA |
| FvH4_2g22530 | FveIAA6-qRT-F | AGTACTAAGAAGAGGGCTTCAC |
|  | FveIAA6-qRT-R | CGTTTTTCCGGTAAGATCTGAT |
| FvH4_2g28970 | FveIAA29-qRT-F | GCAAGAGGCAGAAGAAAAGAGA |
|  | FveIAA29-qRT-R | TGGAAATTATGGTGGGCATGG |
| FvH4_7g28670 | FveGA20ox1-qRT-F | CGGATCAAAGCTCCTCCAATG |
|  | FveGA20ox1-qRT-R | CTCATTCCCAGAAGCTCCAT |
| FvH4_2g33690 | FveSLY1-qRT-F | AGGAGGACGATTACAAGATGAAG |
|  | FveSLY1-qRT-R | TAGTGTTCTGGCATCAACGTG |
| FvH4_6g04910 | FveCKX5-qRT-F | CTCTTCTTTTCTGTTCTTGGCG |
|  | FveCKX5-qRT-R | CGTGTTGACATAATCGAACGAG |
| FvH4_2g39230 | FveCKX8-qRT-F | CTATTTGTACTTGTCAGTCGGC |
|  | FveCKX8-qRT-R | GCTCTGCGTTTCTCTCTTCT |
| FvH4_6g46500 | FveDWARF-qRT-F | CAGTCTTTGGGGAAACCACAG |
|  | FveDWARF-qRT-R | GCTTCGTTCATTAGGATGTATCTG |
| FvH4_1g22040 | FveMYB10L-qRT-F | ATGGAGGTGAGAAAAGGTTC |
|  | FveMYB10L-qRT-R | GTTTCCCAAAAGCTTCCGAAG |
| FvH4_1g22020 | FveMYB10-qRT-F | TGGCATCATGTTCCTCTCAAA |
|  | FveMYB10-qRT-R | CTCTGCAAACTCTCCTCTCTTG |
| FvH4_7g01160 | FveCHS1-qRT-F | AAGTCTGCAGCTAATGGGCACAAG |
|  | FveCHS1-qRT-R | ACTCGAATGAACCCGGATGCCTTA |
| FvH4_2g39520 | FveDFR2-qRT-F | CCGGACTTTCGCCTCTATTT |
|  | FveDFR2-qRT-R | GCTTTCGGATGCTCGTACA |
| FvH4_5g01170 | FveANS-qRT-F | GAAGTGCGTACCCAACTCCATCGT |
|  | FveANS-qRT-R | ACCTTCTCCTTGTTGACGAGCCC |
| FvH4_7g33840 | FveUFGT-qRT-F | TTTGGTTCGGTGCTCATA |
|  | FveUFGT-qRT-R | AGCATCTGTCCCATCTGGT |
| FvH4_1g27460 | RAP -qRT-F | CAAGTTCCAGCAATCGAAGA |
|  | RAP -qRT-R | TGGGAAGGATCACAAGTTGA |
| FvH4_2g35050 | FveGA20ox4-qRT-F | CTCAGTCAACTGCCTCGATTT |
|  | FveGA20ox4-qRT-R | CAGACTCAGGTTCTTGGACATAC |
| FvH4_2g20740 | FveCESA4-qRT-F | ACTTGAAGGACAAGGTTCATCCG |
|  | FveCESA4-qRT-R | CGAGTGTTGTTACCAGGCCAT |
| FvH4_1g00670 | FveCESA7-qRT-F | GAGCCCCTGAGATGTACTTCTGT |
|  | FveCESA7-qRT-R | ATGGTGTCCCATCTTGCATTATCC |
| FvH4_3g07420 | FveCESA8-qRT-F | GCACCTGAGTTCTACTTCTCACAG |
|  | FveCESA8-qRT-R | GGTGTTCCATCTTGCATAGTCCA |
| FvH4_3g42760 | FveCSLE1-qRT-F | GCCGAGATCACCATTTGCTT |
|  | FveCSLE1-qRT-R | ATCTTACTTCTTCCGGAATCCGG |
| FvH4_2g02970 | FveXTH10-qRT-F | GGACAGATTGACATGCAAATAAAGC |
|  | FveXTH10-qRT-R | GTTGATCCTCTCTTCACGGTTGT |
| FvH4_6g10010 | FveEXP8-2-qRT-F | CAACCTCTGCCCACCTAATC |
|  | FveEXP8-2-qRT-R | GTGAACCTTATGCCTCCTTTCT |
| FvH4_4g24420 | FveGAPDH2-qRT-F | CCCAAGTAAGGATGCCCCCATGTTCG |
|  | FveGAPDH2-qRT-R | TTGGCAAGGGGAGCAAGACAGTTGGTAG |
| AT3G18780 | AtActin2-qRT-F | TCCCTCAGCACATTCCAGCA |
|  | AtActin2-qRT-R | GATCCCATTCATAAAACCCCAGC |
| **Primers used for genotyping** | | |
| FvH4_4g19750 | Forward | GGTTGGATCAACGCTGCGT |
|  | Reverse | GAACGTCACAACACCCCACT |
| FvH4_4g06060 | Forward | GAATCCAGAGCATGCTCAAACCCT |
|  | Reverse | CACCGCCAACTTCATGATGTCAAC |
| FvH4_1g16610 | Forward | GATTCCAAACAGCCGCATTG |
|  | Reverse | ATCCAGAGCACCCATGGAAA |
| FvH4_1g17790 | Forward | CATGTTTGTAGTTGTTACATTG |
|  | Reverse | GTATGAATAGTATTCTCCCAAAT |
| FvH4_1g26640 | Forward | TCAGATGTGATCTGAATCCAC |
|  | Reverse | CTAGAAACCTTAACGGTGAC |
| FvH4_4g07420 | Forward | AGATTGGAGGAAGCTGGAG |
|  | Reverse | GCTAGCCACTCTTTTCCATCA |
| FvH4_4g16660 | Forward | TAGGAGTGGCCAAGAAATTGA |
|  | Reverse | CTCCAAATGAAGTGACTTTTGT |
| FvH4_4g16900 | Forward | TCTATCTCTCTCTAGCTCAGGTT |
|  | Reverse | ATTTACCACATAGGAGAAAACTCC |
| FvH4_4g19400 | Forward | CTGTTGCTGATATTGCATCCA |
|  | Reverse | GTTGCGGTTTGACCAATTAAAT |
| FvH4_4g20560 | Forward | GATTTGGAAAAGGTATAGCCT |
|  | Reverse | CTCAAGAAATTTTGACACATATACAT |
| GFP | Forward | GGCAAGCTGACCCTGAAGTTCAT |
|  | Reverse | TTGTGGCGGATCTTGAAGTTCACC |
